# Nrf2 Signaling Contributes to the Neuroprotective Effects of Urate against 6-OHDA Toxicity

**DOI:** 10.1371/journal.pone.0100286

**Published:** 2014-06-24

**Authors:** Ning Zhang, Hai-Yang Shu, Tingting Huang, Qi-Lin Zhang, Da Li, Guan-Qun Zhang, Xiao-Yan Peng, Chun-Feng Liu, Wei-Feng Luo, Li-Fang Hu

**Affiliations:** 1 Department of Neurology, Jiangsu Key Laboratory of Translational Research and Therapy for Neuro-Psycho-Diseases and The Second Affiliated Hospital of Soochow University, Soochow University, Suzhou, Jiangsu, China; 2 Institute of Neuroscience, Soochow University, Suzhou, Jiangsu, China; 3 Department of Pharmacology, Soochow University, Suzhou, Jiangsu, China; Florey Institute of Neuroscience and Mental Health, The University of Melbourne, Australia

## Abstract

**Background:**

Mounting evidence shows that urate may become a biomarker of Parkinson's disease (PD) diagnosis and prognosis and a neuroprotectant candidate for PD therapy. However, the cellular and molecular mechanisms underlying its neuroprotective actions remain poorly understood.

**Results:**

In this study, we showed that urate pretreatment protected dopaminergic cell line (SH-SY5Y and MES23.5) against 6-hydroxydopamine (6-OHDA)- and hydrogen peroxide- induced cell damage. Urate was found to be accumulated into SH-SY5Y cells after 30 min treatment. Moreover, urate induced NF-E2-related factor 2 (Nrf2) accumulation by inhibiting its ubiquitinationa and degradation, and also promoted its nuclear translocation; however, it did not modulate Nrf2 mRNA level or Kelch-like ECH-associated protein 1 (Keap1) expression. In addition, urate markedly up-regulated the transcription and protein expression of γ-glutamate-cysteine ligase catalytic subunit (γ-GCLC) and heme oxygenase-1 (HO-1), both of which are controlled by Nrf2 activity. Furthermore, Nrf2 knockdown by siRNA abolished the intracellular glutathione augmentation and the protection exerted by urate pretreatment.

**Conclusion:**

Our findings demonstrated that urate treatment may result in Nrf2-targeted anti-oxidant genes transcription and expression by reducing Nrf2 ubiquitination and degradation and promoting its nuclear translocation, and thus offer neuroprotection on dopaminergic cells against oxidative stresses.

## Introduction

Parkinson's disease (PD) is the second most common neurodegenerative disorder. It is pathologically featured by dopaminergic neuron losses in substantia nigra and the formation of cytoplasmic inclusion bodies, although its etiology remains elusive. Dopamine replacement therapy remains the first line strategy in PD treatment; however, its effectiveness fades with disease progression. Therefore, more potential targets are needed to be identified to expand the therapeutic strategies.

Urate constitutes the end product of purine metabolism in primates due to the lack of urate oxidase, which catalyzes the conversion of urate to allantonin. Relatively higher levels of urate exist in human plasma. The reference ranges of urate in clinical practices are typically 3.4–7.2 mg/dl (200–430 µmol/l) for men and 2.4–6.1 mg/dl for women (140–360 µmol/l) [1]. However, its concentration in human brain (∼30 µmol/l) is much lower than in blood. In 1994, a post-mortem study revealed that urate was lower in the nigrostriatal tissue of PD patients compared with age-matched controls [2]. Subsequently, several epidemiological studies consistently showed that higher but still normal level of plasma urate was associated with lower risk and slower progression of PD [3] Urate also favored the outcomes of non-motor symptoms of PD and other neurodegenerative disorders [4]. All these lines of evidence suggest a beneficial role of urate in PD. In recent years, our and other groups' work in both *in vitro* and *in vivo* PD models substantiated the neuroprotective actions of urate [5]–[7]. However, the cellular and molecular mechanisms were poorly understood.

Recent studies identified that nuclear factor E2-related factor 2 (Nrf2) was strongly induced in nucleus of PD nigral neurons [8]. Nrf2 is a transcription factor regulating the expression of antioxidant response elements (ARE) contained genes such as heme oxygenase-1 (HO-1), NAD(P)H quinone oxidoreductase-1, glutathione-S-transferases and other glutathione synthesizing enzymes [9]. Alterations in Nrf2 signaling were linked to abnormal redox homeostasis. Loss of Nrf2-mediated transcription exacerbated the vulnerability of dopaminergic neurons to oxidative stresses [10]. Nrf2 knockout mice showed a greater loss of dopaminergic neurons compared with wild type mice when exposure to 1-methyl-4-phenyl-1,2,3,6-tetrahydropyridine (MPTP) [11]. Therefore, Nrf2 may serve as a critical signaling molecule in the neuroprotective strategies agaisnt PD pathogenesis. In the present study, we sought to examine whether Nrf2 signaling is involved in the protective effect of urate on dopaminergic cells. Our findings demonstrated that urate could activate Nrf2 transactivity by inhibiting its ubiquitination and degradation without disrupting Nrf2 association with Kelch-like ECH-associated protein 1 (Keap1), and thus protected dopaminergic cells (SH-SY5Y and MES23.5) against oxidative insults.

## Materials and Methods

### Reagents and antibodies

Uric acid and 6-hydroxydopamine (6-OHDA) were purchased from Sigma-Aldrich (St Louis, MO, USA). Cycloheximide (CHX) was obtained from Beyotime (Nantong, China) and lipofectinamine 2000 from Invitrogen (Carlsbad, CA, USA). The antibodies against γ-glutamate-cysteine ligase catalytic subunit (γ-GCLC), γ-glutamate-cysteine ligase modifier (γ-GCLM), HO-1 and Nrf2, Keap1, ubiquitin were purchased from Abcam (New Territories, Hong Kong) and Santa Cruz (California, USA), respectively. Other primary antibodies were obtained from Cell Signaling Technology (Boston, MA, USA). All reagents for cell culture were obtained from Life technologies (Van Allen Way, Carlsbad, USA).

### Cell culture and treatment

Undifferentiated SH-SY5Y cells were purchase from ATCC and cultured in Dulbecco's modified Eagle's Medium (DMEM) supplemented with 10% fetal bovine serum and 1% penicillin/streptomycin in a 5% CO_2_ atmosphere at 37°C. MES23.5 cells (kindly provided by Prof. Wei-dong Le, Institute of Health Science, Shanghai Institutes For Biological Sciences, CAS) were cultured in DMEM/F12 growth medium supplemented with 5% fetal bovine serum, 2 mmol/L glutamine and Sato's chemically defined medium to a final concentration of 5 mg/ml insulin, 5 mg/ml transferrin, 48.6 mg/ml pyruvic acid, 6.3 ng/ml progesterone, 5 ng/ml sodium selenite and 4 mg/ml putrescine [12].

### Cell viability measurement

Cell viability was determined as previously described [5]. In brief, at the end of treatment, culture medium was replaced with the medium containing 3-(4,5-Dimethylthiazol-2-yl)-2,5-diphenyltetrazolium bromide (MTT) at a final concentration of 0.5 mg/ml and cells were incubated at 37°C for 4 h. After that, culture supernatant was carefully removed. The insoluble formazan was then dissolved in dimethyl sulphoxide. The absorbance was determined at 570 nm with the reference wavelength at 630 nm using a microplate reader (TECAN M200 Pro, Grodig, Austria).

### Urate measurement

Urate was assessed with an assay kit from Cayman Chemical (Ann Arbpr, MI, USA) according to the manufacturer's instructions. In brief, SH-SY5Y cells were treated with 200 µmol/l urate. After incubation for indicated time periodst, the culture supernatants were collected for extracellular urate assay. Cells were then washed twice with phosphate-buffered saline (PBS) and harvested in a solution of 150 mmol/l phosphoric acid. After centrifugation at 15,000 g for 15 min at 4°C, the resulting supernatants were used for intracellular urate determination. For urate assay, 20 µl samples were mixed with 30 µl assay buffer in a 96-well plate, followed by the addition of 50 µl reaction mixture that contains the probe and enzyme mix. The mixtures were then incubated at 37°C for 30 min. Fluorescence was measured at Ex/Em = 535/590 nm in a microplate reader (TECAN, GmbH, Austria). The intracellular urate level was normalized by the protein level and expressed as µmol/g protein. The protein concentrations were determined using the BCA kit (Pierce Chemical, Rockford, IL, USA).

### Protein carbonyl determination

For protein carbonyl assay, SH-SY5Y cells were cultured in 100 mm dishes until they reached confluence. Cells were harvested by centrifugation at 12,000 rpm for 5 min. The pellets were resuspended in 300 µl ice-cold PBS and sonicated. The supernatants were then collected for protein carbonyls assay using a commercial ELISA kit (BioCell Corp, New Zealand). The carbonyl level was normalized by the protein concentration and expressed as nmol/mg protein.

### Immunoblotting and immunoprecipitation

Whole lysates were prepared by washing cells twice with chilled PBS and homogenized in lysis buffer (150 mM NaCl, 25 mM Tris (pH7.5), 5 mM EDTA, 1% Nonidet P-40 and protease inhibitor cocktail tablets (Roche Diagnostics, Penzberg, Germany). Protein samples were boiled for 5 min prior to separation on 10% sodium dodecyl sulfate-polyacrylamide gel and transferred onto polyvinylidene fluoride membranes (Millipore, Bedford, MA, USA). Membranes were then blocked and incubated with primary antibodies against proteins of interest at 4°C overnight with gentle shaking. Afterwards, membranes were briefly washed and incubated with HRP-conjugated second antibodies (Jackson Laboratory, USA). The results were visualized by ECL chemiluminescence (GE healthcare, Buckinghamshire, UK). The band densities were quantified by Image J software (National Institute of Health, USA).

For immunoprecipitation (IP), cells were lysed in lysis buffer as mentioned above. Cell lysates were precleared with protein A/G Plus-agarose beads (Santa Cruz, California, USA) and incubated with 5 µg of the affinity-purified antibody overnight at 4°C. The lysates were then precipitated by incubation with protein A/G-agarose beads at 4°C for 2 h. After washing with RIPA buffer three times, immunoprecipitation complexes were eluted in sample buffer by boiling for 5 min and subjected to immunoblotting as described above. When measuring ubiquitinated Nrf2, proteins extracts were denatured first to disrupt the potential association of Nrf2 with other proteins before IP.

### Confocal imaging

Cells were fixed in 4% paraformaldehyde and permeabilized in PBS with 0.1% Triton X-100 for 5 min. Next, coverslips were blocked in 3% bovine serum albumin/PBS for 1 h. After that, coverslips were incubated with anti-Nrf2 antibodies at 4°C overnight, followed by incubation with Alexa Fluor 488 chicken anti-rabbit IgG (Molecular Probes, Eugene, OR, USA) for another 1 h. Subsequently, coverslips were mounted onto slides with mounting medium containing DAPI. Cells were observed and scanned under a confocal microscope (LSM 700, Zeiss, Germany).

### Cytosolic and nuclear fraction lysate preparation

The cytosolic and nuclear fractions were separated with a kit (Beyotime institute of Biotechnology, China) according to the manufacturer's instructions. In brief, cells were harvested and lysed with 100 µl cytosolic extract A reagent containing 1 mM PMSF and vortexed for 5 seconds. The lysates were then added with 5 µl cytosolic extract B reagent and violently vortexed for another 5 seconds. After that, the lysates were centrifuged at 13,000 g at 4°C for 5 min and the supernatants were collected and designated as the cytosolic fractions. The resulting pellets were added with 30 µl nuclear extract reagent containing 1 mM PMSF and vortexed for 15–30 seconds every 2 min interval during a period of 30 min, and centrifuged again at 13,000 g at 4°C for 10 min. The resulting supernatants were extracted as nuclear proteins.

### Reverse Transcription PCR

RNA was extracted using TRIzol reagent (Invitrogen, Carlsbad, CA, USA). Equal amounts of RNA (1 µg) were reversely transcribed into cDNA using cDNA synthesis kit (Fermentas). An equal volume of cDNA product was amplified using PCR Master Mix kit (Fermentas) with primers (Genscript, Nanjing, China) as listed: human GCLC [NM_001498.3] (forward 5′-TGA GAT TTA AGC CCC CTC CT-3′ and reverse 5′-TTG GGA TCA GTC CAG GAA AC-3′); GCLM [NM_002061.2] (forward 5′-TTT GGT CAG GGA GTT TCC AG-3′ and reverse 5′-ACA CAG CAG GAG GCA AGA TT-3′); HO-1 [NM_002133.2] (forward 5′-CCT AAA CTT CAG AGG GGG CG-3′ and reverse 5′-ATG GCT CAA AAA CCA CCC CA-3′); Nrf2 [NM_006164.4] (forward 5′-TTC AAA GCG TCC GAA CTC CA-3′ and reverse 5′-AAT GTC TGC GCC AAA AGC TG-3′) and β-actin [NM_001101.3] (forward 5′-AAG AGA GGC ATC CTC ACC CT-3′ and reverse 5′-TAC ATG GCT GGG GTG TTG AA-3′). PCR products were separated in a 2% agarose gel and stained with Gel view. The band densities were analyzed with Image J software.

### Transient transfection with siRNA targeting Nrf2

The small interfering RNA (siRNA) oligonucleotides targeting human Nrf2 (si-Nrf2-1: sense 5′-CCC GUU UGU AGA UGA CAA UTT -3′; antisense 5′-AUU GUC AUC UAC AAA CGG GTT-3′; si-Nrf2-2: sense 5′-GCC CAU UGA UGU UUC UGA UTT-3′; antisense 5′-AUC AGA AAC AUC AAU GGG CTT -3′) and nonspecific oligonucleotides were ordered from GenePharma (Shanghai, China). SH-SY5Y cells were transiently transfected with siRNAs using Lipofectamine 2000 (Invitrogen, Carlsbad, CA, USA) when reaching 70–80% confluence. The Nrf2 knockdown efficiency was determined at 24 h post-transfection with immunoblotting.

### Intracellular glutathione measurement

Glutathione assays were performed with a kit according to the manufacturer's instructions (Jiancheng Biochemical Reagent Co. Nanjing, China). Briefly, cultures were washed with ice-cold PBS and deproteinated with 10% trichloroacetic acid. After centrifugation, the supernatants were mixed with working buffer containing glutathione reductase, DTNB and total glutathione amortization buffer and incubated for 5 min, followed by the addition with 0.16 mg/ml NADPH and incubation for 25 min. Subsequently, the absorbance was measured at 450 nm using a microplate reader as described above. Total glutathione content was determined with a standard curve obtained from the defined concentrations of reduced glutathione.

### Statistical analysis

All data were presented as mean ± SEM. Statistical differences were assessed with one-way analysis of variance followed by a post hoc (Tukey) test for multiple group comparison. Differences with *P*<0.05 were considered statistically significant.

## Results

### Urate pretreatment alleviated the 6-OHDA-induced injury to dopaminergic cells

**Figure 1:**
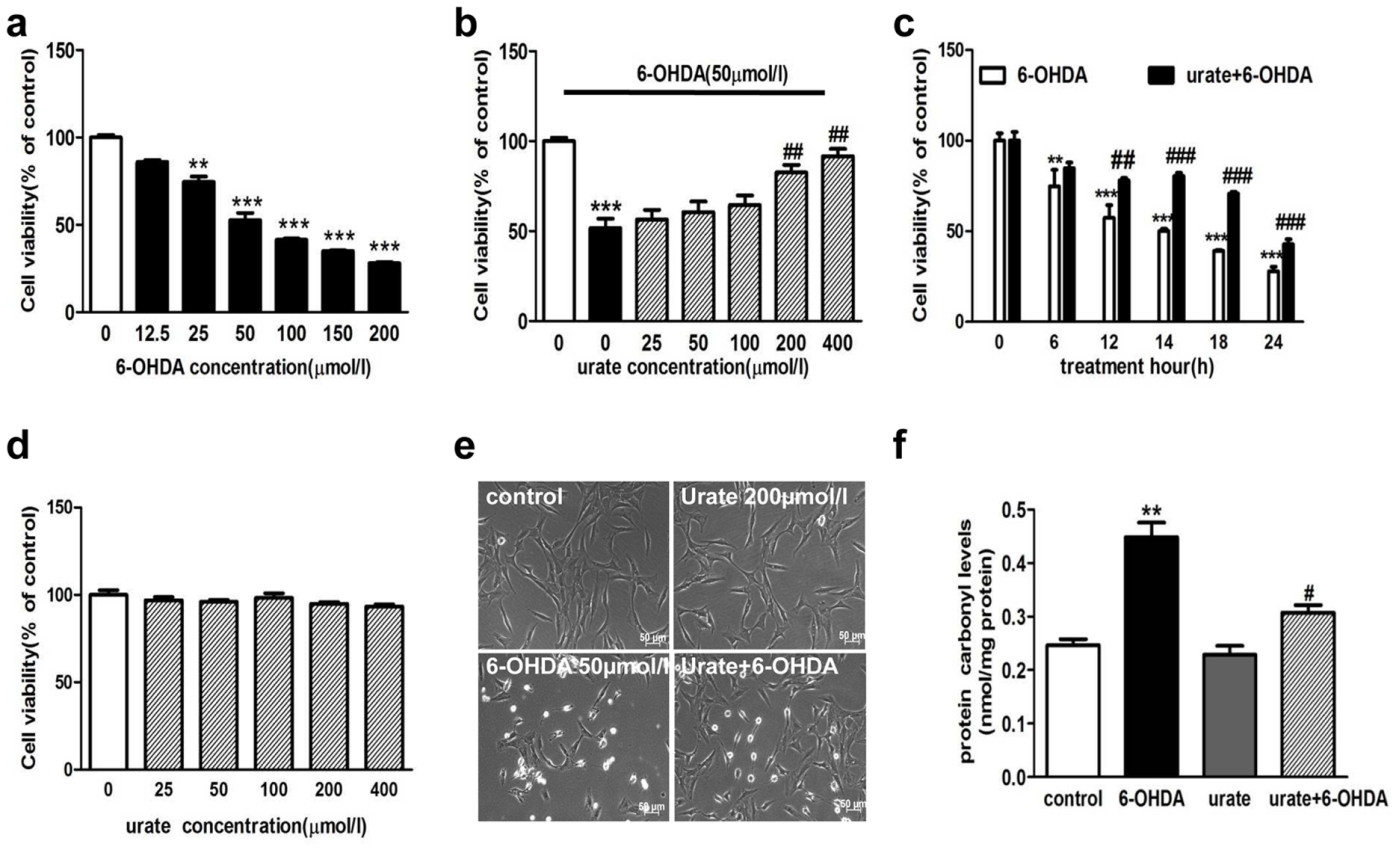
Effect of urate pretreatment on 6-OHDA-induced injury in SH-SY5Y cells. (**a**) Concentration-dependent toxicity of 6-OHDA in SH-SY5Y cells. Cells were treated with various concentrations of 6-OHDA for 14 h. Cell viability was then determined by MTT method. (**b**) Effect of urate on 6-OHDA-induced cytotoxicity. Cells were pre-treated with urate (25, 50, 100, 200 and 400 µmol/l) for 30 min, followed by 50 µmol/l 6-OHDA treatment for 14 h. (**c**) Effect of 200 µmol/l urate pretreatment on cell survival when cells were exposed to 50 µmol/l 6-OHDA for different time periods. (**d**) Effect of urate treatment for 24 h on SH-SY5Y cell viability at indicated concentrations. (**e**) Morphological changes of SH-SY5Y cells when cells were treated with 50 µmol/l 6-OHDA or 200 µmol/l urate, or in combination with both for 14 h. (**f**) Cells were pretreated with 200 µmol/l urate for 30 min followed by 50 µmol/l 6-OHDA for 14 h. The protein carbonyl levels were determined as described in methods. Data were presented as Mean ± SEM. N = 6 for each group in cell viability assay and the results were repeated three times independently. **P<0.01, ***P<0.001 versus controls; #*P*<0.05, ##*P*<0.01, ###*P*<0.001 versus 6-OHDA group.

6-OHDA is a commonly used toxin for inducing PD-like models in both *in vivo* and *in vitro* studies. In this study, we observed that 6-OHDA treatment resulted in a significant decrease of cell viability in human dopaminergic neuroblastoma cell line (SH-SY5Y) in concentration- and time-dependent manners (Fig. 1a,c). 50 µmol/l 6-OHDA treatment for 14 h reduced the cell viability by 47.3% as compared to controls. We then evaluated the effect of urate (25, 50, 100, 200 and 400 µmol/l) on 6-OHDA-induced damage in SH-SY5Y cells. It was found that urate pretreatment for 30 min appeared to attenuate the 6-OHDA-induced toxicity in a concentration-dependent manner. Specifically, at 200 µmol/l and 400 µmol/l, urate increased the cell viability by 37.4% and 43.5% as compared to 6-OHDA-treated group (Fig. 1b). Moreover, 200 µmol/l urate pretreatment was still able to protect against the cytotoxicity when cells were exposed to 50 µmol/l 6-OHDA for up to 24 h, although the protective effect became less significant than that at earlier time points (Fig. 1c). To preclude the possibility that urate caused any toxicity to SH-SY5Y cells after 24 h incubation, cells were treated with urate alone and the cell viability was determined at 24 h later. The results showed that urate, at tested concentrations (25, 50, 100, 200 and 400 µmol/l), did not produce any toxic effect on SH-SY5Y cells (Fig. 1d). In addition, we observed that SH-SY5Y cells exhibited short spiny neurite-like processses in vehicle-treated group. After exposure to 50 µmol/l 6-OHDA for 14 h, most cells shrank and cell processes disappeared. The morphological changes were markedly alleviated in urate-pretreated group (Fig. 1e). Considering that 200 µmol/l urate, which was within its physiological range, produced obvious protection against 6-OHDA-induced toxicity, 200 µmol/l urate was then applied in the studies herein reported.

### Urate could be accumulated into dopaminergic cells

**Figure 2:**
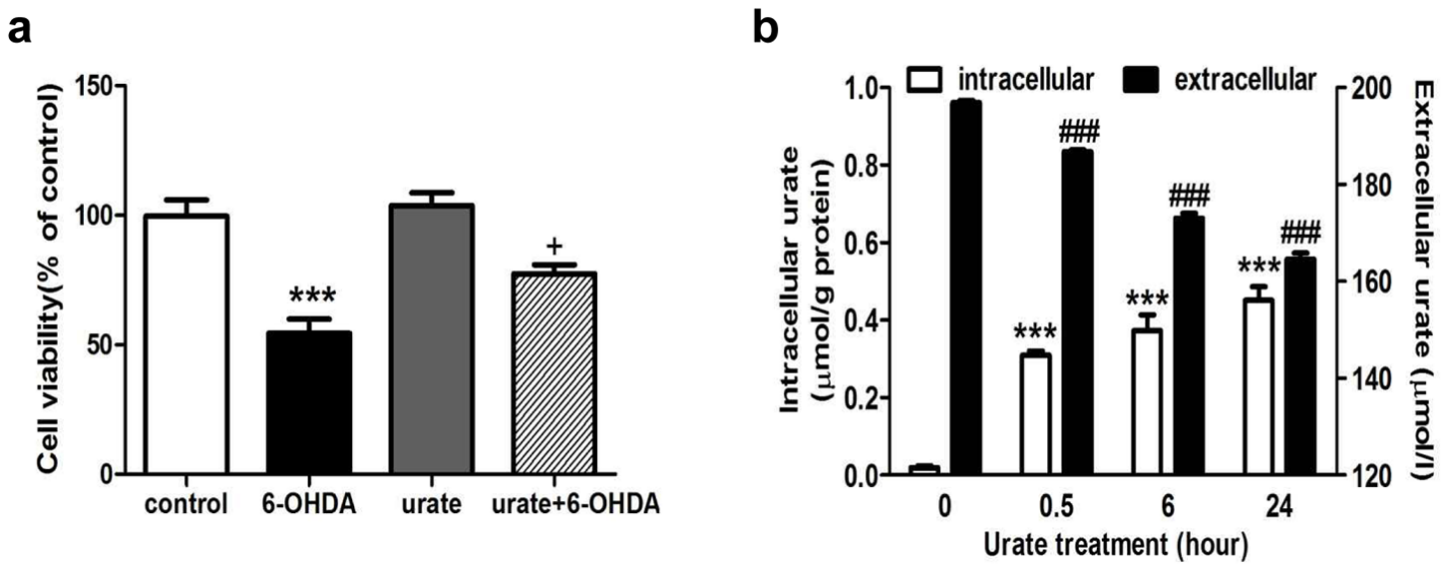
Urate was accumulated into SH-SY5Y cells and exerted protection intracellularly. (**a**) Effect of urate pre-treatment on cell viability. Urate was removed by washout after 30 min pre-incubation, followed by 6-OHDA treatment for 14 h. (**b**) Accumulation of urate into SH-SY5Y cells as incubation time increased. Cells were treated with 200 µmol/l urate for different time periods as indicated. The extracellular and intracellular urate content was determined as described in “Material and methods”. The intracellular urate level was normalized by the protein concentration. Results were shown as Mean ± SEM, n = 3. +*P*<0.05 versus 6-OHDA group; ***P<0.001 versus controls or intracellular value of controls (0 h); *###P*<0.001 versus extracellular value of controls (0 h).

Interestingly, we observed urate's protection remained even if urate was washed out before 6-OHDA exposure (Fig. 2a). It is less likely that this protection was a false positive observation resulted from the direct interaction between urate and 6-OHDA in the extracellular milieu. Next, we assessed whether urate could be transported into SH-SY5Y cells. To achieve this, cells were treated with 200 µmol/l urate for 0.5, 6 and 24 hours. The urate levels in culture supernatant and cell lysates at these time points were then determined and defined as extracellular and intracellular urate content, respectively. It was observed that intracellular urate level increased in a time-dependent manner whilst the extracellular counterpart decreased (Fig. 2b). At 0.5 h after urate addition, the extracellular urate level decreased to 186.75 µmol/l while the intracellular urate reached 0.31±0.01 µmol/g protein, which was about 15 folds over the basal level (0.02±0.01 µmol/g protein). Furthermore, no significant amount of urate was detected in the culture supernatant at another 24 h later when extracellular urate was removed after 24 h incubation (data not shown), implying reverse transportation of urate may not exist in this cell line.

### Urate protected against H_2_O_2_-induced cell damage to dopaminergic cells

As urate was previously reported to act as a pro-oxidant under some conditions [13], we also studied the effect of urate on oxidative stress by determining the protein oxidation product carbonyl levels. We found that 50 µmol/l 6-OHDA treatment enhanced the cellular protein carbonyl levels, which was obviously attenuated in the presence of 200 µmol/l urate pretreatment. Furthermore, urate treatment alone did not affect the protein carbonyl levels, as shown in Fig. 1f.

**Figure 3:**
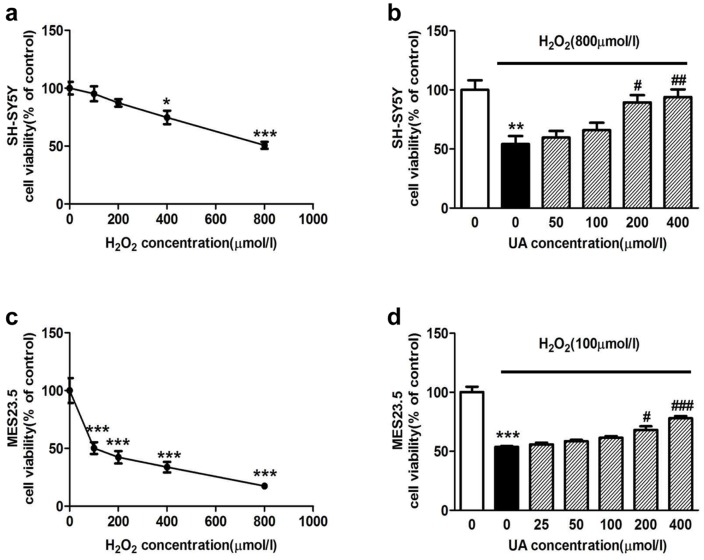
Effect of urate on H_2_O_2_-induced cell damage in SH-SY5Y and MES23.5 cells. (**a, c**) Concentration-dependent toxicity of H_2_O_2_ treatment for 12 h to SH-SY5Y (**a**) and MES23.5 cells (**c**). (**b, d**) Cells were pretreated with urate (25, 50, 100, 200 and 400 µmol/l) for 24 h, followed by H_2_O_2_ treatment at the indicated concentration in SH-SY5Y (**b**) and MES23.5 (**d**) cells. The results were repeated three times. *P<0.05, **P<0.01, ***P<0.001 versus controls; #*P*<0.05, ##*P*<0.01, ###*P*<0.001 versus H_2_O_2_ group.

To determine whether urate was protective against other oxidative insults, SH-SY5Y cells and another dopaminergic cell line (MES23.5) were subjected to H_2_O_2_ treatment (100, 200, 400 and 800 µmol/l) for 12 h. As can be seen from Fig. 3a,c, H_2_O_2_ resulted in a differential toxicity to SH-SY5Y (Fig. 3a) and MES23.5 (Fig. 3c) cells. Specifically, 800 µmol/l H_2_O_2_ treatment reduced the cell viability by 49.3% in SH-SY5Y whilst merely 100 µmol/l H_2_O_2_ decreased it by 49.8% in MES23.5 cell line. Pretreatment with urate at 200 µmol/l and 400 µmol/l for 24 h markedly increased the cell viability as compared to H_2_O_2_-treated group in SH-SY5Y cells (Fig. 3b). Similar trend was observed in MES23.5 cells (Fig. 3d).

### Urate suppressed Nrf2 ubiquitination and degradation

**Figure 4:**
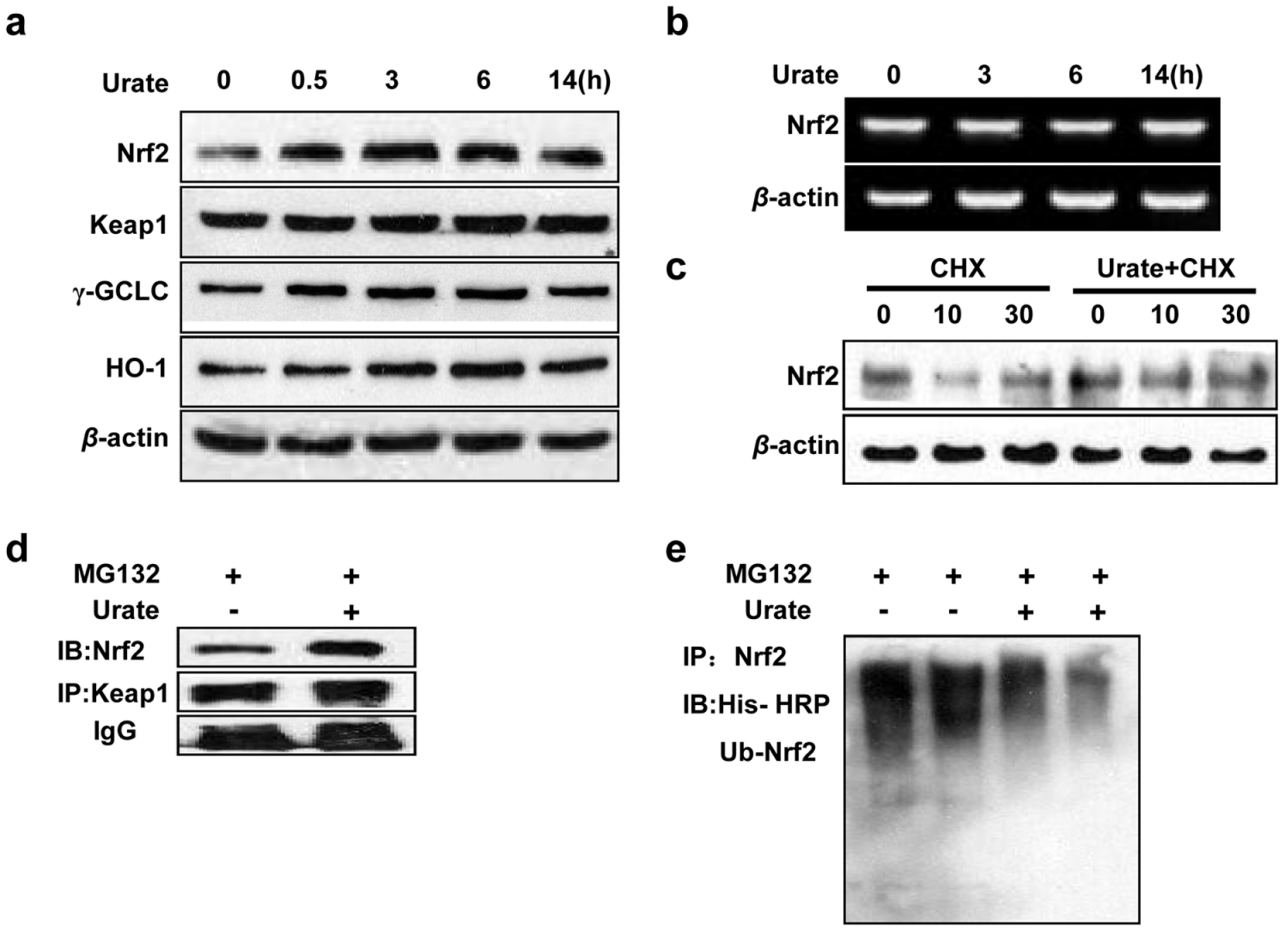
Effect of urate on Nrf2 activation in SH-SY5Y cells. (**a, b**) Immunoblotting and reserve transcription PCR analysis showing the protein and mRNA levels of Nrf2, Keap1, γGCLC and HO-1.Cells were treated with 200 µmol/l urate for 0.5, 3, 6 and 14 hours. *β*-actin served as loading controls. (**c**) Effect of urate on Nrf2 protein level in the presence of 1 µg/ml CHX. Cells were harvested and lysed at 0, 10 and 30 min after CHX addition with or without urate treatment. (**d**) Urate did not disrupt Keap1-Nrf2 complex. Cells were treated with 200 µmol/l urate for 6 h. The association of this complex was assessed using IP with anti-Keap1, followed by immunoblotting with anti-Nrf2. (**e**) Urate inhibited Nrf2 ubiquitination. Cells were treated with or without urate for 6 h in the presence of MG132 (25 µM). For detecting ubiquitinated Nrf2, samples were subjected to IP with anti-Nrf2, followed by IB with an anti-His-HRP-conjugated antibody. The results were independently repeated at least three times.

We then explored the signaling mechanisms that contributed to the protection of urate. Nrf2 is a master regulator against oxidative stress and it controls the transcription of several anti-oxidant genes. We observed that 200 µmol/l urate treatment markedly elevated the protein levels of Nrf2 and its control genes including *γ-gclc* and *ho-1*. The elevation, detected as early as 0.5 h after treatment, lasted at least 6 h and came to decline at 14 h later (Fig. 4a). Notably, the Nrf2 transcription was not altered by urate (Fig. 4b). Moreover, we observed in the presence of CHX, a protein translation inhibitor, Nrf2 protein level decreased rapidly. About 50% of the total Nrf2 was decreased at 10 min after CHX addition. However, this decrease appeared much slower in the presence of urate (Fig. 4c), implying urate may delay Nrf2 degradation.

Nrf2 protein is rapidly degraded by the 26S proteasome with its half-life at approximately 15 min [14], [15]. Keap1, known as Nrf2 repressor, is crucial for its rapid turnover and functions as an adaptor for Nrf2 ubiquitination [16], [17]. Therefore, we processed to study the protein level of Keap1 and its interaction with Nrf2 via immunoprecipitation. We observed no significant change in Keap1 expression after urate treatment for up to 14 h. Of interest, we detected a significant increase in Nrf2 level in Keap1 immunoprecipitates from urate-treated cells in the presence of proteasome inhibitor MG132 (Fig. 4d). Furthermore, urate markedly reduced the ubiquitination of Nrf2, as shown in Fig. 4e.

### Urate induced Nrf2 accumulation and its nuclear translocation in dopaminergic cells

**Figure 5:**
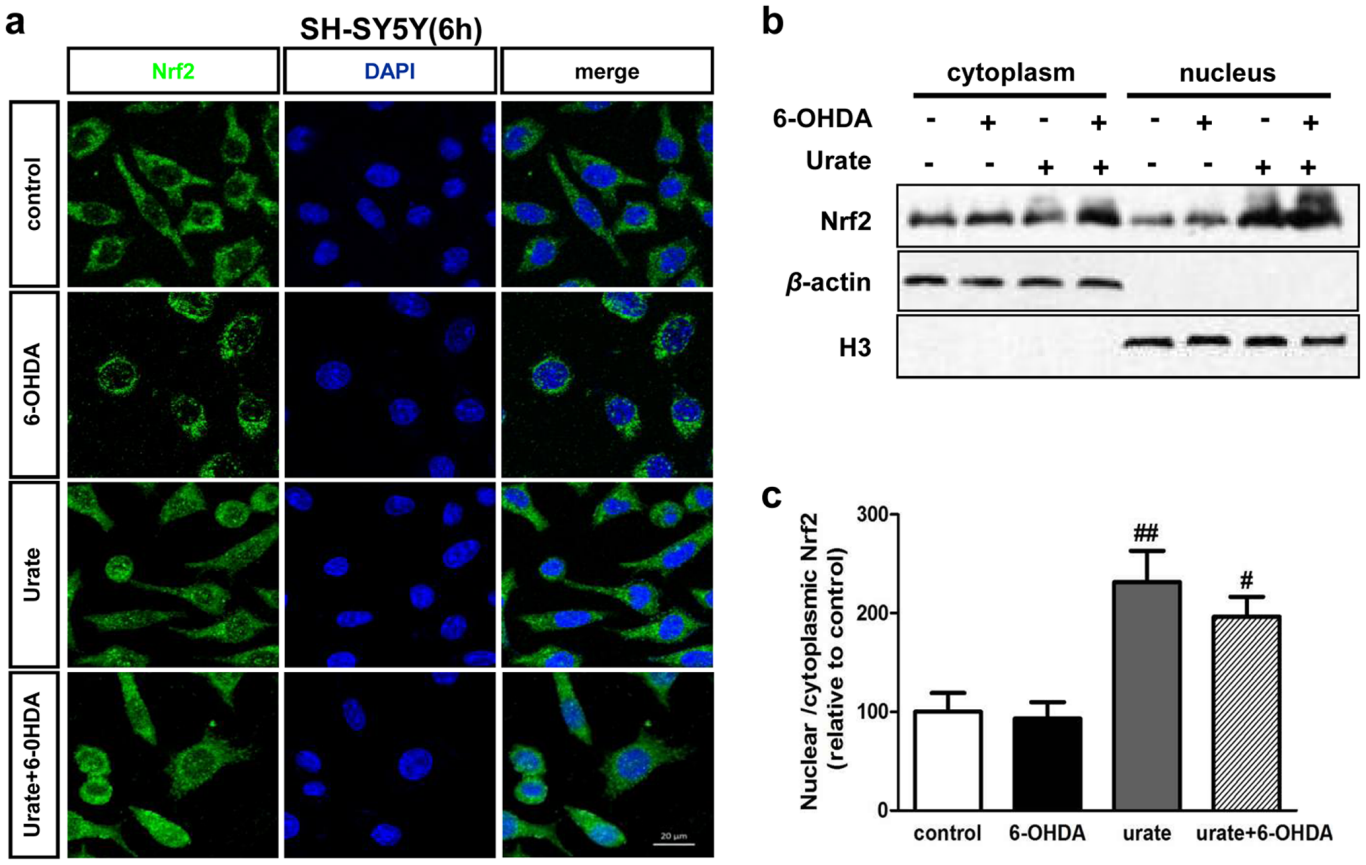
Effect of urate on the subcellular distribution of Nrf2 in SY-SY5Y cells. Cells were pre-incubated with urate for 30 min prior to 6-OHDA treatment for 6 h. (**a**) Representative pictures showing the subcellular distribution of Nrf2 (FITC/green) in SH-SY5Y cells. Nuclei were stained with DAPI (blue). Scale bar = 20 µm. (**b, c**) Cytoplasmic and nuclear fractions were prepared and subjected to immunoblotting analysis. H3 and *β*-actin were used for nuclear and cytoplasmic protein markers, respectively. Mean ± SEM, n = 4. #*P*<0.05 versus 6-OHDA group; ##*P*<0.01 versus control.

Nrf2 controls and initiates the transcription of oxidation-related genes such as *γ-gcl* and *ho-1* once it accumulates and translocates into the nucleus [18]. Therefore, the subcellular distribution of Nrf2 was also studied by confocal scanning in combination with immunoblotting. We observed Nrf2 was mostly distributed in cytoplasm in both control and 6-OHDA (50 µmol/l, 6 h) -treated cells. However, it was mainly localized to the nucleus with urate pretreatment (Fig. 5a). This redistribution was most prominent in urate alone treated cells. To verify these observations, cytosolic and nuclear compartments of SH-SY5Y cells were fractioned and subjected to immunoblotting (Fig. 5b). The results showed Nrf2 protein was present at higher levels in the nuclear fraction than that in the cytosolic after urate treatment (Fig. 5c), implying urate may promote Nrf2 nuclear translocation. The phenomenon was validated in another dopaminergic cell line MES23.5. Similarly, the pictures showed that Nrf2 was predominantly located in the cytoplasm of control and 6-OHDA (50 µmol/l, 6 h)-treated cells. There was an obvious Nrf2 accumulation in the nuclei of urate-treated cells, which was more prominent in the cells without 6-OHDA treatment (Fig. S1a). We also extended our study to 14 h after 50 µmol/l 6-OHDA treatment. The results showed that similar trends of Nrf2 re-distribution were observed in SH-SY5Y cells with urate pre-treatment, although the changes were not as obvious as those at 6 h after treatment (Fig. S1c).

### Urate promoted the transcription and protein expression of Nrf2-target genes

**Figure 6:**
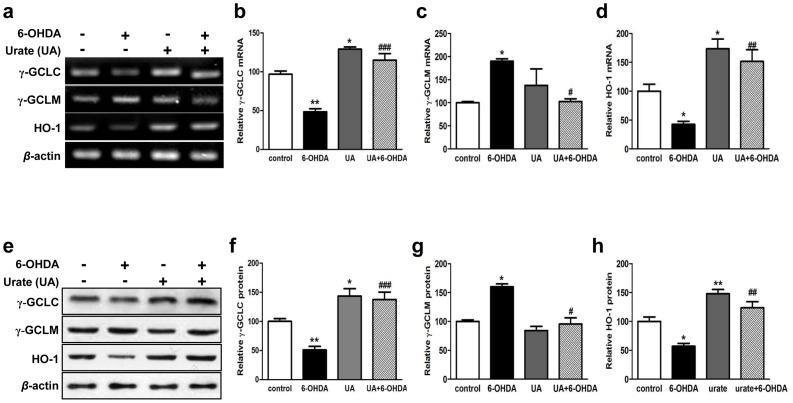
Effects of urate on the mRNA and protein expression of the antioxidant genes. Cells were pretreated with 200 µmol/l urate or vehicle, followed by 6-OHDA treatment for 14 h. The mRNA (**a-d**) and protein (**e-h**) levels of *γ-gclc*, *γ-gclm* and ho-1 were assessed by reserve transcription PCR and immunoblotting, respectively. *β*-actin served as loading controls. Group data were obtained by normalizing to *β*-actin and expressed as percentage of control values. Mean ± SEM, n = 3. *P<0.05, **P<0.01 versus control; #*P*<0.05, ##*P*<0.01, ###*P*<0.001 versus 6-OHDA group.

Next, we continued to study the mRNA and protein expression of Nrf2-regulated genes including *γ-gclc*, *γ-gclm* and *ho-1*. 200 µM urate treatment for 14 h was found to enhance the mRNA and protein expression of *γ-gclc* and *ho-1* in SH-SY5Y cells (Fig. 6a, e). In contrast, the transcription and translation of *γ-gclm* was not obviously altered by urate treatment. In addition, we observed the mRNA levels of *γ-gclc* and *ho-1* were dramatically decreased while that of *γ-gclm* was increased in response to 6-OHDA treatment. However, the transcriptional changes of these genes were significantly reversed in the presence of urate pretreatment (Fig. 6b-d). Similar trends were observed at the protein levels (Fig. 6f-h). Urate treatment was able to elevate the expression of γ-GCLC and HO-1 protein in both vehicle- and 6-OHDA- treated cells. Meanwhile, it alleviated the increase of γ-GCLM protein expression caused by 6-OHDA in SH-SY5Y cells.

### Nrf2 knockdown abolished the protection of urate on SH-SY5Y cells

**Figure 7:**
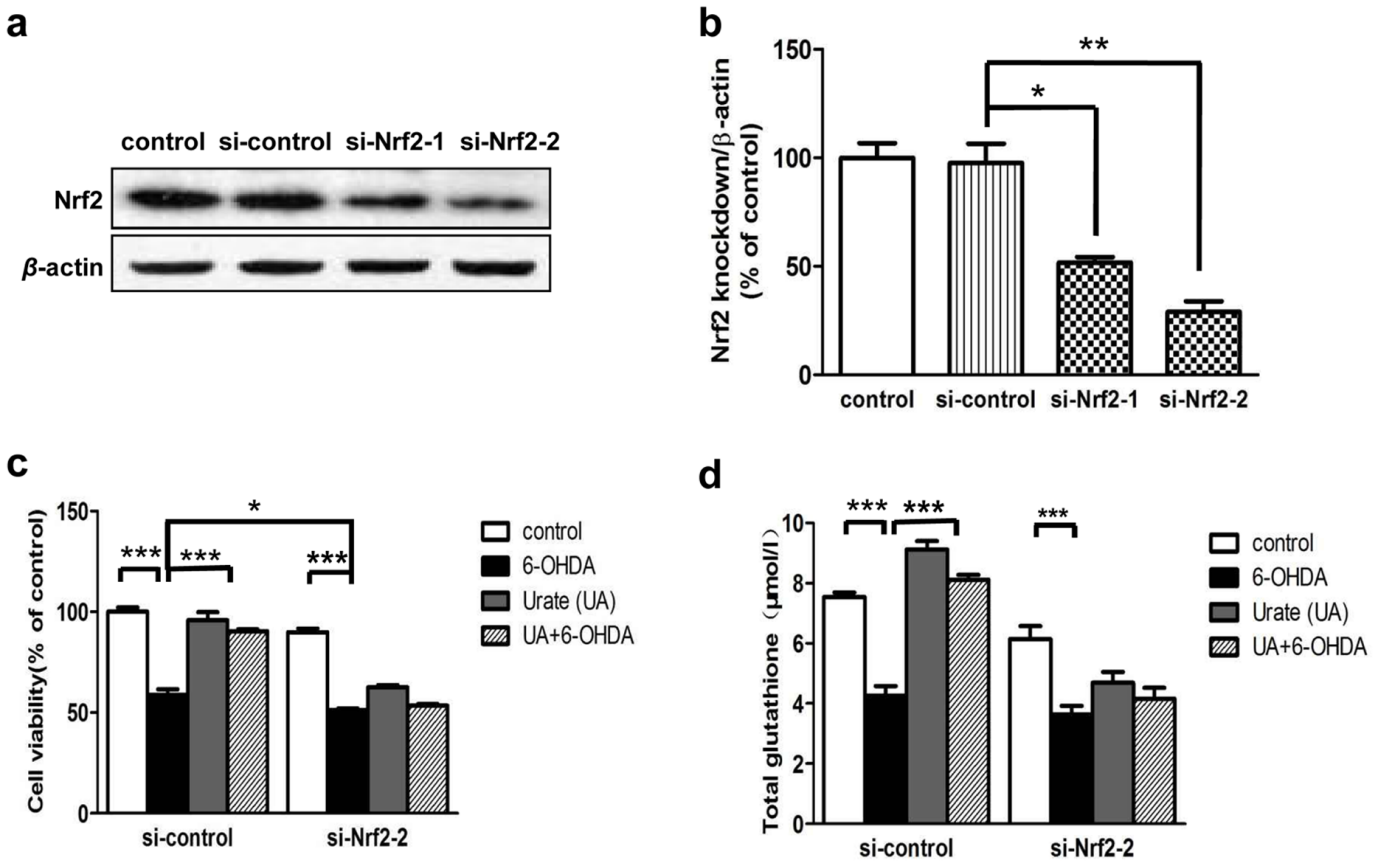
Nrf2-siRNA eliminated the protective effects of urate. (**a, b**) SH-SY5Y cells were transfected with scrambled siRNA (si-control) or two different siRNAs targeting human Nrf2 (si-Nrf2-1 and si-Nrf2-2). RNA interference efficiency was determined by immunoblotting at 24 h later. Mean ± SEM, n = 3. *P<0.05, **P<0.01 versus control group. (**c, d**) Cells were exposed to 6-OHDA (50 µmol/l) for 14 h, with or without urate pretreatment at 24 h after transfection. Cell viability (**c**) and intracellular glutathione level (**d**) were measured as described above. Results were presented as mean ± SEM, n = 3. *P<0.05, **P<0.01, ***P<0.001.

To substantiate the role of Nrf2 in urate's protection, the effects of urate on cell survival and glutathione level were determined following introduction of Nrf2 siRNA into SH-SY5Y cells. Immunoblotting revealed that individual transfection with two different siRNAs against human Nrf2 (si-Nrf2-1, si-Nrf2-2) successfully reduced Nrf2 protein expression at 24 h post-transfection, as compared to both untransfected- (control) and mocked transfected (si-control) cells (Fig. 7a,b). The knockdown efficiency of si-Nrf2-2 appeared to be more obvious than that of si-Nrf2-1. Therefore, si-Nrf2-2 was taken in the following study.

We observed that Nrf2 siRNA-transfected cells were more susceptible to 6-OHDA-induced toxicity (12.7% reduction compared with control siRNA transfected cells, *P*<0.05). More importantly, Nrf2 knockdown abolished the beneficial effects offered by urate pretreatment on 6-OHDA-injured cells (Fig. 7c). The elevation of total glutathione stimulated by urate pre-treatment was abolished in Nrf2 siRNA-transfected cells (Fig. 7d).

## Discussion

**Figure 8:**
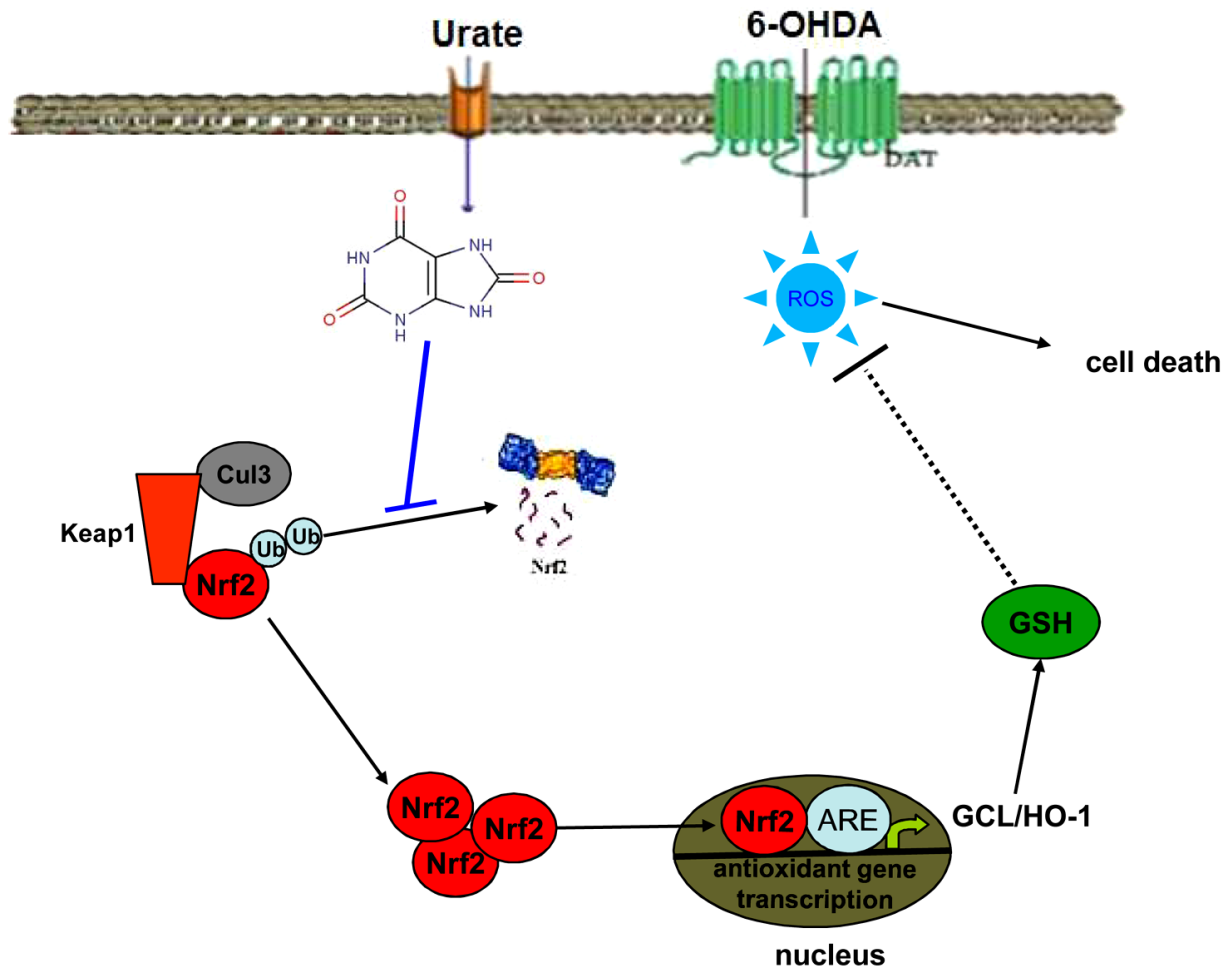
Schematic representations illustrating how urate may activate Nrf2 and thus protects against oxidative stresses. Urate may be accumulated into cells via undefined transporters. It led to Nrf2 accumulation by inhibiting its ubiquitination and degradation and promoted Nrf2 translocate into nuclei, where Nrf2 may transactivate the transcription of antioxidant enzyme genes such as GCL, HO-1, and thus protected against oxidative stresses in dopaminergic cells.

Urate has been proposed as a neuroprotectant candidate for PD. However, the mechanisms that underlie urate's neuroprotection remains poorly understood. In this study, we showed that urate pretreatment protected dopaminergic cells against 6-OHDA- and H_2_O_2_- induced damage. This protection and its elevation on intracellular glutathione level was markedly abated by knockdown of Nrf2 with siRNA. We also demonstrated that urate could be accumulated into SH-SY5Y cells and exerted the protective effect intracellularly. Furthermore, we provided the evidence that urate induced Nrf2 accumulation by inhibiting its ubiquitination and degradation, and promoted Nrf2 translocation into nuclei, where it transactivated the transcription and translation of Nrf2 target genes including *γ-gclc* and *ho-1* (summarized in Fig. 8). However, urate did not modulate Nrf2 mRNA and Keap1 protein levels, nor did it disrupt Nrf2-Keap1 association. Therefore, our findings demonstrated for the first time that Nrf2 signaling may contribute to the protection of urate on dopaminergic cells.

Urate exists at relatively lower concentrations in the brain and are inversely related to the risk of PD. The urate levels were found to be decreased in the brains of PD patients [2]. All these indicate a beneficial effect of urate on dopaminergic neuron and PD progression. However, it remained unclear whether or not urate could be accumulated into dopaminergic neurons. Our results showed the accumulation of urate into SH-SY5Y cells. This is in line with a previous report by Cipriani *et al*. [19].But, Guerreiro S *et al*. claimed that urate could not be significantly accumulated into mesencephalic neuron [6].This discrepancy may be explained by the difference in sensitivity of the analytic methods applied. The fluorescence-based assay with urate was used in this study. This may be more sensitive than the spectrophotometric assay. Urate may be transported into dopaminergic cells via some kind(s) of unidentified transporters. Notably, urate transporters are highly expressed in kidney and brain [20].

Moreover, we demonstrated a direct protection offered by relatively high concentrations of urate (≧200 µmol/l) against 6-OHDA-induced toxicity on dopaminergic cells. This is consistent with some previous reports. However, there are also studies reporting that pretreatment with urate at 0–100 µmol/l tended but failed to significantly decrease H_2_O_2_-induced cell death in MES23.5 cells and demonstrating that urate's neuroprotection was astrocyte-dependent [19], [21]. To substantiate our finding, we also examined if urate protected against H_2_O_2_-induced damage in both SH-SY5Y and MES23.5 cells. The results consistently showed that urate tended to yield protective effects at lower concentrations (<200 µM), and significantly increased the cell viability in H_2_O_2_-treated dopaminergic cells at higher levels (≧200 µmol/l) (Fig. 3). Thus, both direct and indirect neuroprotection of urate may exist; however, relatively higher urate levels may be required for its direct neuroprotection. Stimulation and release of astrocyte-derived neurotrophic factor(s), although still undefined, may considerably amplify the neuroprotection of urate.

Nrf2 is a critical transcription factor defending against oxidative stress. It controls and initiates the transcription of several oxidation-related genes such as *γ-gcl* and *ho-1* [18]. Our present study showed that urate promoted Nrf2 accumulation and nuclear translocation by inhibiting its ubiquitination and degradation. It also enhanced the transcription and protein expression of Nrf2 target genes including *γ-gclc* and *ho-1*, both of which are closely related to redox homeostasis. Furthermore, Nrf2 knockdown was observed to abolish the protection offered by urate and its stimulation on glutathione. All these evidence supports a critical role of Nrf2 signaling in urate's neuroprotection. Our previous results showed that in 6-OHDA-lesioned rat model of PD, urate's neuroprotection was linked with Akt/GSK3β signaling pathway. Many studies showed that PI3K/Akt signaling pathway was associated with Nrf2 activation and glutathione synthesis [22]. Therefore, it is likely that the Nrf2 activation was secondary to the action of urate on Akt/GSK3β signaling.

Nrf2 is primarily controlled at protein level due to the presence of two degradation domains (degrons) within its Neh2 and Neh6 regions [23].The Neh2 domain contains a redox-sensitive degron that interacts with the redox sensor Keap1 to allow ubiquitination and subsequent degradation of Nrf2 by a Cullin3-Rbx1 complex under unstressed conditions [24], [25]. Our results showed urate increased the Nrf2 protein levels; however, it did not affect Nrf2 gene transcription and Keap1 protein expression, implying urate may enhance Nrf2 stability at protein level. It has been widely accepted that chemical activation of Nrf2 results from Nrf2 dissociation from Keap1, thereby allowing Nrf2 to escape from Keap1-mediated proteasomal degradation. However, we found urate inhibited Nrf2 ubiquitination without disrupting Nrf2-Keap1 association. This is consistent with a recent study reporting the mechanisms by which 5,6-dihydrocyclopenta-1,2-dithiole-3-thione (CPDT) and sulforaphane activated Nrf2 [26]. It is possible that urate induced conformational change of Keap1 and thus rendered Keap1-bound Nrf2 unreachable by the ubiquitin ligase. We will assess this possibility in our future study.

Glutathione is a major intracellular antioxidant. Depletion of glutathione pools is involved in PD development. Our results showed that urate enhanced the transcription and expression of the rate-limiting enzymes for glutathione biosynthesis and elevated the intracellular glutathione content in 6-OHDA-treated SH-SY5Y cells. Nrf2 knockdown attenuated urate's protection and its effect on glutathione, implying Nrf-2 activation was involved in the increase of glutathione stimulated by urate. However, it should be noted that other mechanisms that contributed to the glutathione elevation may also exist. For example, urate was reported to promote cysteine uptake and enhance glutathione levels in SH-SY5Y cells and hippocampus slice cultures [27].

Interestingly, in this study 6-OHDA treatment did not elicit an obvious anti-oxidant response in cells. We examined the Nrf2 distribution at both 6 h and 14 h after 6-OHDA exposure with immunofluorescent staining and immunoblotting. We did not observed a marked nuclear translocation of Nrf2 in 6-OHDA treated cells. The intracellular glutathione level was also reduced. The occurrence of anti-oxidant response may be dependent upon the severity of cell injury. A previous study showed that 6-OHDA injection into rat striatum enhanced nuclear Nrf2 translocation, which occurred at earlier time points (0.5 h and 1 h) after lesion and gradually returning to the basal level at 4 h later [28]. This indicates a compensatory anti-oxidant response to the toxic insult may be induced but only detected at early time period. Long-term exposure to toxic insult may inhibit the compensatory/resistance responses. Of note, 6-OHDA increased the mRNA level of *γ-gclm*, which is a modifier subunit of γ-GCL. In most cases, the catalytic subunit accounts for the activity of an enzyme. Therefore, the increase of *γ-gclm* mRNA probably occurs in compensation for the *γ-gclc* reduction. Despite of this, urate treatment differentially regulated the transcription of these two subunits of γ-GCL protein in 6-OHDA-treated cells. It is likely that the increase of γ-GCLC protein is sufficient for glutathione synthesis elevation since it is one of the most readily induced anti-oxidant genes and rate-limiting for its synthesis.

In fact, the antioxidant activity of urate is complex. It possesses antioxidant properties comparable to those of ascorbate and provides most of the antioxidant capacity in human fluid [29], [30]. In this study, we found urate not only alleviated 6-OHDA-induced toxicity, but also protected against H_2_O_2_-induced toxicity. Therefore, it is more likely a general antioxidant reagent. Although it acts as a powerful scavenger of peroxynitrite, peroxide and hypochlorous acid, urate does not react with some oxidants such as superoxide. It also shows iron-chelating activity independent of its direct antioxidant actions. Notably, urate acts as a pro-oxidant in some circumstances [31], [32]; however, this possibility is excluded in this study, as urate treatment did not increase protein carbonyls in SH-SY5Y cells.

In sum, this study demonstrated that urate activated Nrf2 by inhibiting its ubiquitination and degradation, and thus protected against oxidative insults to dopaminergic cells.

## Supporting Information

Figure S1 **Urate induced Nrf2 protein accumulation and its translocation from cytoplasm to nucleus in SH-SY5Y cells (14 h) and MES23.5 cells (6 h).** Cells were pre-incubated with 200 µmol/l urate for 30 min prior to 50 µmol/l 6-OHDA treatment for 6 h (MES23.5 cell) or 14 h (SH-SY5Y cells). (**a,c**) Representative images showing the subcellular distribution of Nrf2 (FITC/green) in MES23.5 cells (a) and SH-SY5Y cells (c). Nuclei were stained with DAPI (blue). Scale bar = 20 µm. (b,d) Immunoblotting analysis of Nrf2 in nuclear and cytoplasmic fractions of cells subjected to abovementioned treatments. H3 and *β*-actin were used for nuclear and cytoplasmic protein markers, respectively.
(TIF)
