# Supplementary figures and images for: Nrf2 Signaling Contributes to the Neuroprotective Effects of Urate against 6-OHDA Toxicity
Source: PLoS One. 2014 Jun 24;9(6):e100286. doi: 10.1371/journal.pone.0100286 (PMC4069024; doi:10.1371/journal.pone.0100286)

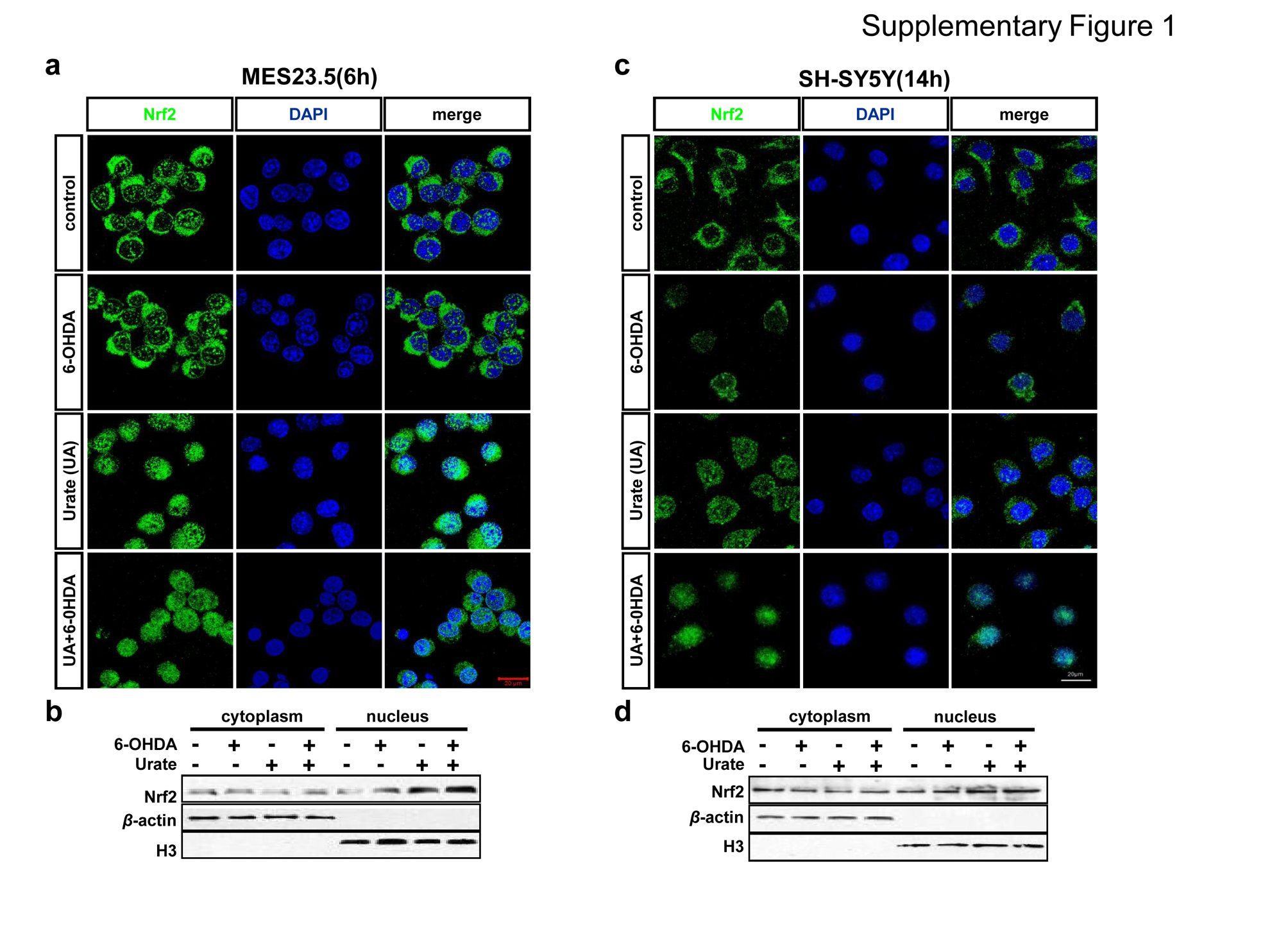

Supplement: Figure S1 — Urate induced Nrf2 protein accumulation and its translocation from cytoplasm to nucleus in SH-SY5Y cells (14 h) and MES23.5 cells (6 h). Cells were pre-incubated with 200 µmol/l urate for 30 min prior to 50 µmol/l 6-OHDA treatment for 6 h (MES23.5 cell) or 14 h (SH-SY5Y cells). (a,c) Representative images showing the subcellular distribution of Nrf2 (FITC/green) in MES23.5 cells (a) and SH-SY5Y cells (c). Nuclei were stained with DAPI (blue). Scale bar = 20 µm. (b,d) Immunoblotting analysis of Nrf2 in nuclear and cytoplasmic fractions of cells subjected to abovementioned treatments. H3 and β-actin were used for nuclear and cytoplasmic protein markers, respectively. (TIF) [file pone.0100286.s001.tif]
